# Breast Cancer Patients’ Parenting Concerns User Personas: A Qualitative Analysis

**DOI:** 10.3390/curroncol33090501

**Published:** 2026-08-24

**Authors:** Ying Zhang, Ming Liu, Xiaoning Fan, Hailing Tu, Yin Wang, Jingfang Hong

**Affiliations:** 1 School of Nursing, Anhui Medical University, No. 81 Mei Shan Road, ShuShan District, Hefei 230032, China; 2445011304@stu.ahmu.edu.cn (Y.Z.);; 2 The First Affiliated Hospital of University of Science and Technology of China, Hefei 230001, China; 3 Nursing International Collaboration Research Center of Anhui Province, Hefei 230601, China; 4 The First Affiliated Hospital of Anhui Medical University, No. 218 Ji Xi Road, ShuShan District, Hefei 230022, China

**Keywords:** breast cancer, parenting concerns, precision supportive care, user personas, qualitative research

## Abstract

Breast cancer disproportionately affects young women raising minor children. Unresolved parenting concerns can impair patients mental health, reduce treatment adherence, and compromise long-term oncological outcomes. However, previous studies have paid limited attention to the heterogeneity of parenting concerns in this population and lack a theory-grounded classification framework for stratified supportive care. This descriptive qualitative study, framed by social role theory, recruited 17 Chinese breast cancer patients through maximum variation sampling and conducted semi-structured in-depth interviews. From four dimensions—role expectation, role load, role resources, and role adaptation—the study extracted four distinct parenting concern personas: role overload, role strain, role resilience, and role disablement. The four subgroups differed significantly in maternal role expectations, treatment-related physical burden, family support resources, and coping strategies. These findings can assist clinicians in identifying the specific types of distress faced by different patient subgroups, thereby providing a basis for developing stratified and individualized precision support strategies.

## Simple Summary

Many young breast cancer patients are responsible for caring for their minor children, yet their parenting concerns are often overlooked in routine oncology care. Identifying different groups of mothers with distinct parenting concerns is a prerequisite for delivering precision support; however, previous studies have largely treated parenting concerns as a single variable and seldom distinguished between mothers facing different difficulties. Grounded in social role theory, this study conducted semi-structured in-depth interviews with 17 young Chinese breast cancer patients and identified four distinct parenting concern personas. Notably, current clinical care lacks differentiated support strategies tailored to these personas. The findings highlight the key characteristics and challenges of each persona, emphasizing the importance of providing individualized support to different patient subgroups.

## 1. Introduction

Breast cancer is the most common malignancy and the leading cause of cancer-related mortality among women worldwide, with approximately 2.3 million new cases globally in 2022 and incidence rates continuing to rise [1,2]. Some young breast cancer patients aged 20–55 years are raising minor children, requiring them to simultaneously manage anti-tumor treatment and childcare, thus assuming the dual roles of patient and mother [3,4,5].

Compared with other malignancies, breast cancer impedes patients’ fulfillment of maternal functions in multiple ways. Body image changes resulting from mastectomy lead patients to avoid physical contact with their children [6]; axillary lymph node dissection requires avoiding heavy physical activities such as holding young children to reduce the risk of lymphedema, directly limiting parent–child physical interaction [7]; and surgical damage to breast tissue causes permanent impairment of breastfeeding capacity [8]. These disease-specific treatment-related sequelae persistently trigger guilt and weaken maternal identity, thereby giving rise to parenting concerns [3,5,9,10,11,12]. Parenting concerns are defined as the persistent psychological distress experienced by mothers with cancer arising from worries about their children’s psychological development, quality of daily care, and the perceived inadequacy of spousal or other caregivers [13].

Unresolved parenting concerns can trigger a cascade of negative consequences: decreased parenting self-efficacy and quality of life, heightened psychological distress and persistent physical fatigue, and significantly impaired treatment adherence [14,15]; some patients even delay or forego standard anti-tumor treatment to prioritize childcare, significantly worsening long-term oncological prognosis [16]. Meanwhile, family stress arising from maternal role imbalance disrupts established household division of labor, exacerbates marital conflict, weakens family cohesion [17,18,19], and increases the risk of emotional and behavioral problems in children [20].

In clinical practice, parenting concerns among patients raising minor children exhibit marked group heterogeneity [21]. However, existing studies have predominantly employed variable-centered homogeneous statistical analyses that overlook individual differentiation in maternal role experiences and lack theoretically grounded subtyping frameworks, making it difficult to guide stratified, individualized precision supportive care in oncology settings.

Social role theory posits that individual behavior is guided by socially prescribed role expectations; when individuals face resource constraints or capacity limitations that make it difficult to fulfill these expectations, role conflict and role overload ensue [22]. For breast cancer patients raising minor children, the dual identities of “patient” and “mother” often generate competing demands—the illness impairs their ability to fulfill maternal functions, and patients become trapped in role conflict as they struggle to balance treatment and childcare, manifesting as guilt, self-blame, and helplessness. When family support is insufficient or resources are scarce, role load further intensifies, ultimately undermining their maternal identity and self-efficacy. Thus, this theory provides an appropriate analytical lens for understanding the heterogeneity of parenting concerns in breast cancer patients.

User personas, also termed user profiles or archetypes, are label-based user models constructed by collecting, integrating, and mining user data to accurately reflect the characteristics, needs, and behavioral patterns of target populations, serving as an important approach for delivering personalized and precision services [23]. In healthcare, this method can either build quantitative label models based on large-scale datasets or generate archetypal profiles through team consensus from qualitative interview data. As a precision analytical tool, user personas can intuitively distinguish the heterogeneous needs of different patient populations, providing a theoretical basis for implementing targeted clinical intervention strategies [24].

Grounded in social role theory, this study conducted semi-structured in-depth interviews with breast cancer patients raising minor children to construct distinct parenting concern personas. The findings aim to provide empirical evidence for developing stratified, individualized intervention and support strategies in routine oncology clinical practice, ultimately improving treatment adherence and long-term survival outcomes among breast cancer patients with childcare responsibilities.

## 2. Materials and Methods

### 2.1. Ethical Considerations

Ethical approval was obtained from the Institutional Review Board of Anhui Medical University (Approval No. 81250975, 9 December 2025). This study was conducted in accordance with the principles of the Declaration of Helsinki (World Medical Association, 2024). All participants received written and verbal information regarding the study purpose, procedures, the voluntary nature of participation, and their right to withdraw at any time prior to enrollment, and all provided written informed consent.

### 2.2. Study Design

A descriptive qualitative study design was adopted to achieve the research objectives. This study is reported in accordance with the Consolidated Criteria for Reporting Qualitative Research (COREQ) checklist (Supplementary Materials S1) [25].

### 2.3. Participant Recruitment

This study was conducted between December 2025 and March 2026 at a tertiary general hospital in Hefei, Anhui Province, China. To comprehensively describe patient experiences, purposive sampling following the principle of maximum variation was employed to recruit participants from the Department of Breast Surgery, the Day Ward, and the Department of Medical Oncology, based on variations in age, educational level, and treatment stage. Potential participants meeting the inclusion criteria were identified through electronic medical record review, and eligible patients were subsequently provided with study information and invitations to enroll. The Parenting Concerns Questionnaire (PCQ) [26] was administered to eligible patients to assess their level of parenting concerns. Sample size was determined by data saturation; recruitment ceased when interview data began to repeat and no new themes emerged [27].

Inclusion criteria were:

(1) Pathologically confirmed diagnosis of breast cancer, currently undergoing treatment or in the rehabilitation phase;
(2) Caring for at least one child aged ≤18 years;
(3) Conscious, able to communicate normally, and possessing basic literacy skills.

Exclusion criteria were:

(1) Comorbid severe mental illness, cognitive impairment, or other severe physical illnesses that precluded participation in interviews;
(2) Children in foster care or out-of-home placement, with the patient not assuming primary parenting responsibilities;
(3) Refusal to sign the informed consent form or withdrawal from the study.

A total of 20 eligible participants were recruited, of whom 3 declined to participate (1 due to low mood, 2 due to physical discomfort).

### 2.4. Data Collection

An initial interview guide was developed through literature review and consultation with oncology experts, and refined through pilot interviews with three participants, resulting in the final interview guide (Table 1). Brief introductory communication was conducted before each interview to inform participants of the study purpose, estimated duration, and general topic areas, and to address any questions, thereby reducing participants’ sense of unfamiliarity. Each interview lasted 30–60 min and was conducted in a quiet, uninterrupted conference room. All interviews were conducted by a researcher (Ying Zhang) trained in qualitative research methods. Interviews were audio-recorded throughout, with non-verbal information such as facial expressions, tone of voice, and gestures also documented. The researcher posed follow-up and clarification questions based on participants’ responses. After the interview, participants’ contact information was retained for member checking, and transcripts were returned to participants for confirmation and correction.

### 2.5. Data Analysis

Within 24 h after each interview, two researchers (Ying Zhang and Ming Liu) transcribed the audio recordings verbatim, and two other researchers (Xiaoning Fan and Hailing Tu) verified the transcript data. Two researchers (Ying Zhang and Ming Liu) used NVivo 12 software to extract meaning units related to parenting concerns and assigned preliminary codes. At this stage, 68 initial codes were generated, such as “worrying that children would be left without care after one’s death,” “lack of alternative caregivers,” and “cancer genetic anxiety.” Subsequently, the research team repeatedly compared and categorized the codes, clustering them into sub-themes based on shared characteristics. Guided by social role theory, four core analytical dimensions were ultimately formed: role expectation, role load, role resources, and role adaptation.

For example, Patient P15 described: “After chemotherapy, I have no strength at all. My child pulls my hand wanting me to play with him, but I can’t even hold him.” This was coded as “physical inability to support parenting behaviors,” grouped under the sub-theme “insufficient objective role performance capacity,” and finally assigned to the “role load” dimension. Patient P01 stated: “My child is almost an adult… but I need to let go.” This was coded as “actively adjusting role standards,” grouped under the sub-theme “flexible role cognitive restructuring,” and finally assigned to the “role adaptation” dimension.

Based on differences across the four dimensions, patients with similar role characteristics were grouped together, and data within each group were synthesized to identify heterogeneous patterns of parenting concerns. Four distinct user personas were identified: role overload, role strain, role resilience, and role disablement. Throughout the persona construction process, original data were revisited continuously to ensure that each persona accurately reflected the shared experiences of patients in that subgroup.

To clearly present the heterogeneous characteristics of the different personas, coding labels under each dimension were classified and integrated, and word clouds were generated using the online tool WordArt (https://wordart.com) for visual representation. All word clouds were generated based on keywords extracted from the coding labels under each dimension. The outputs were carefully reviewed and revised by all authors, who take full responsibility for the accuracy of all graphical content presented in this article. In addition, each persona was comprehensively presented through multiple formats, including tabular summaries, narrative abstracts, key feature extraction, and representative quotations.

### 2.6. Rigour and Reflexivity

This study strictly adhered to the qualitative rigour criteria proposed by Lincoln and Guba—namely credibility, dependability, confirmability, and transferability [28]—to ensure research quality.

(1) Credibility: An iterative team analysis strategy was employed, whereby two researchers independently completed coding and then compared results through regular meetings to discuss disagreements until consensus was reached. For investigator triangulation, two researchers participated in data coding and theme development, and a third researcher (Hong Jingfang) was invited to participate in discussion and final adjudication when coding disagreements arose. Interview data were cross-validated with field notes taken during the analysis process to contextualize the findings. Additionally, transcripts were returned to participants for confirmation after interviews (member checking) to ensure that the analytical results were consistent with participants’ authentic experiences.
(2) Transferability: This study provided detailed descriptions of the research context, participant recruitment strategies, inclusion and exclusion criteria, participant demographic and clinical characteristics, and complete data collection and analytical procedures. On this basis, readers and future researchers can assess the applicability and transferability of the findings to their own contexts.
(3) Dependability: A complete audit trail was maintained throughout the research process, including interview audio recordings and verbatim transcripts, NVivo coding data and records of all modifications, coding comparison records, theme development documentation, and all analytical records from initial codes to core dimensions. These materials ensured the transparency and traceability of the research process.
(4) Confirmability: The research team engaged in continuous critical reflection throughout the study, holding regular reflexivity meetings and documenting team discussions and individual reflective journals. These materials were incorporated into the audit trail to minimize the influence of researchers’ preconceptions on analytical conclusions. Coding disagreements were resolved through team discussion, with a third researcher consulted when necessary to reach consensus. The research team comprised one expert with over 20 years of experience in psychosocial oncology (Jingfang Hong, PhD), one expert trained in systematic qualitative research methodology (Yin Wang, PhD), and four master’s-level nursing students trained in qualitative research methods (Ying Zhang, Ming Liu, Xiaoning Fan, Hailing Tu). All team members were female, and interviewers had no prior caregiving relationships with participants. Prior to interviews, participants were clearly informed of the study purpose, and the voluntary nature of participation and freedom to withdraw were emphasized to reduce the influence of social desirability on participants’ responses.

## 3. Results

### 3.1. Characteristics of Participants

A total of 17 participants completed the interview, with each participant being interviewed once. The mean age was 34.0 years (SD = 5.64, range: 26–45 years). The mean total score on the Parenting Concerns Questionnaire (PCQ) was 48.1 ± 10.3 (range: 28–64). Educational levels ranged from middle school to university. Most participants had a disease duration of ≤1 year, with 1 to 3 minor children. The majority were diagnosed with stage II breast cancer. Demographic and clinical characteristics are detailed in Table 2.

### 3.2. User Persona Labelling System for Parenting Concerns in Breast Cancer Patients

Based on qualitative analysis, we constructed four distinct user personas representing different patterns of parenting concerns among breast cancer patients. The four identified personas are presented in Table 3.

#### 3.2.1. Role Overload Type

Narrative Summary:

This type accounted for 41.2% of the sample (n = 7). Their parenting concerns were highly concentrated on daily caregiving for young children, companionship deficits due to insufficient physical strength, and concerns about disease genetic risk. The combination of physical treatment burden and weak family human resources amplified feelings of powerlessness, leading to passive avoidance as the primary coping strategy for parenting conflicts.

Key characteristics:

Maximal role load: Chemotherapy fatigue combined with round-the-clock care demands of young children; physical capacity cannot support basic parenting behaviours. Weak role resources: Low income, absent spousal care, lack of intergenerational support buffer. Moderate role expectations: No perfectionist cognition; guilt stems entirely from objective inability to perform caregiving behaviours. Passive–avoidant adaptation: Parent–child interactions significantly reduced; worries about disease inheritance and children’s developmental deficits.

Illustrative quotations:

*“The doctor only told me to focus on treatment. No one told me how to take care of my child. I’m afraid my condition will worsen and that it will affect my child.”*

*(P02)*

*“I’m not afraid of anything except passing this on to my child.”*

*(P05)*

*“What I worry about most is that if I’m gone, my child is still young—he can’t even dress or feed himself without someone else’s help. No one will love him or teach him.”*

*(P07)*

*“After chemotherapy, I have no strength at all. My child pulls my hand wanting me to play with him, but I can’t even hold him. I feel so sorry for him.”*

*(P15)*

#### 3.2.2. Role Strain Type

Narrative Summary:

This type accounted for 23.5% of the sample (n = 4). Their parenting concerns were highly concentrated on academic guidance for adolescent children, psychological and emotional nurturance, and self-judgment of maternal identity. Driven by internalised perfect mother expectations, they experienced profound self-blame when unable to meet children’s developmental needs, often accompanied by parent–child communication blockage.

Key characteristics:

Excessive role expectations: Deeply ingrained perfect mother cognition; strong sense of self-imposed responsibility for adolescent children’s development. Moderate role load: Physical impairment does not completely prevent caregiving but cannot adequately meet emotional nurturance needs of adolescents. Family resources provide material support only: Lack psychological and parent–child communication assistance. Negative internalising adaptation: Chronic self-attribution; breeding identity-level guilt and uncertainty.

Illustrative quotations:

*“I don’t know how much longer I can live. I’m afraid I won’t be able to help when my child goes to university or looks for a job. I can only worry.”*

*(P10)*

*“My child is in a rebellious phase and needs guidance, but I’ve been receiving treatment the whole time and don’t have much energy to accompany him. I feel I’ve let him down.”*

*(P12)*

*“My child is afraid to worry me and won’t tell me anything. She hides in her room after school. I’m afraid she’ll develop psychological problems, and I’m afraid my illness will affect her future.”*

*(P14)*

#### 3.2.3. Role Resilience Type

Narrative Summary:

This type accounted for 17.7% of the sample (n = 3). Their parenting concerns were highly focused on children’s academic development, long-term independence skills, and concerns about disease inheritance risks. With adequate family intergenerational and economic support, they could flexibly adjust maternal standards and adopt active approaches to balance treatment and childcare.

Key characteristics:

Flexible role expectations: Able to reconstruct the standard of “good mother” and accept limited companionship. Moderate treatment burden: Rich family resources offset parenting stress. Adequate role resources: Middle to high income; spouse and grandparents share parenting and rehabilitation care. Positive reconstructive adaptation: Proactively builds emotional connections; skilled at mobilising family support to buffer stress.

Illustrative quotations:

*“My child is almost an adult. He’s never left us, and I do worry about his future life. But I need to let go.”*

*(P01)*

*“In the days following chemotherapy, I’m very tired and can’t take care of her. But she’s very independent, so I feel reassured.”*

*(P03)*

*“My child is taking the college entrance exam next year. I’m afraid my condition will fluctuate, but I can’t do anything about it—all I can do now is focus on my treatment.”*

*(P08)*

#### 3.2.4. Role Disablement Type

Narrative Summary:

This type accounted for 17.6% of the sample (n = 3). Their parenting concerns were concentrated on daily care of young children, family financial pressure, lack of care support, and difficulties accessing support information. Multiple resource deficits, combined with multiple parenting responsibilities, left them only able to passively endure various parenting-related stresses.

Key characteristics:

Ordinary role expectations: No extreme perfectionist maternal demands. Role load: Short-term disease impact, combined with young childcare. Complete absence of role resources: Low income, chronic spousal absence, zero intergenerational support, no information support channels. Passive–helpless adaptation: No external buffering forces; multiple practical pressures combined to form complex parenting concerns.

Illustrative quotations:

*“Treatment cost so much money, and the family has almost no income. I can barely afford my child’s school fees. I feel so useless—I can’t get better and I can’t raise my child well.”*

*(P06)*

*“After I got sick, no one could help me with the children. During chemotherapy, I had to leave them with neighbours. I worried they wouldn’t take good care of them. … Even after I got sick, I still had to take care of all three of them just like before.”*

*(P09)*

*“I want to get treatment and take good care of my child, but I don’t know what to do. I asked the doctor at the hospital, but he didn’t have much time to talk to me about these things. No one gives me guidance.”*

*(P11)*

## 4. Discussion

Grounded in role theory, this study adopted a person-centred approach to identify four distinct parenting concern personas among breast cancer patients—namely role overload, role strain, role resilience, and role disablement—and revealed the interplay of maternal cognitive expectations, disease and parenting stress burdens, family structure and intergenerational support resources, and coping styles on parenting concerns. This study distinguished the group heterogeneity of parenting concerns among breast cancer patients, providing evidence for stratified, precision clinical interventions.

The findings of this study extend social role theory. Previous role theory research has largely focused on single role conflicts (e.g., work-family conflict) or role stress in specific contexts, with limited attention to the systematic differentiation of multiple roles in chronic illness contexts [29,30]. This study found that among patients sharing the dual identities of “patient” and “mother,” the manifestations of role conflict differed: some presented as role overload (insufficient objective capacity to fulfill role expectations), some as role strain (excessive subjective expectations leading to self-negation), some as role resilience (supported by adequate resources), and some as role disablement (characterised by comprehensive resource depletion). This finding suggests that role distress in illness contexts is not a unitary phenomenon but manifests in differentiated patterns based on varying combinations of role expectation intensity, role load magnitude, role resource availability, and role adaptation styles. This provides empirical evidence for refining the concepts of “role conflict” and “role load” within social role theory and also suggests that future research on role distress in chronic illness should simultaneously examine the interaction between objective load and subjective expectations.

Most children of patients with the role overload type were infants and toddlers aged 0–6 years, a period when children rely heavily on their mothers for daily physical care. These patients underwent intensive chemotherapy accompanied by severe physical exhaustion, which largely prevented them from fulfilling basic maternal caregiving duties. Consequently, their primary worries revolved around existential fears such as disease progression and the prospect of their children being left without maternal care; younger children corresponded to greater levels of parenting distress, a result consistent with the findings of Zhang et al. [5]. According to attachment theory, this phenomenon reveals that mothers of young children perceive their own existence and their children’s basic welfare as inseparable [31]. Of particular note, many patients expressed fear of cancer inheritance, yet current clinical consultations do not adequately address this need [32,33]. The difficulties of this patient group stem from the temporal and physical conflict between intensive treatment and high-intensity parenting tasks. Parenting concerns can be alleviated through task substitution—for example, during peak chemotherapy fatigue, family members could take over high-energy parenting tasks (such as feeding, bathing, and nighttime soothing) to allow patients necessary physical recovery time. After chemotherapy-related fatigue subsides, patients could be guided to adopt low-energy parent–child interaction methods such as bedside companionship, gentle conversation, and shared picture-book reading, transforming guilt and helplessness into controllable positive parenting behaviours [34].

The children of patients with the role strain type were predominantly in adolescence—a developmental stage characterised by heightened needs for emotional companionship, psychological guidance, and developmental support [35]. In interviews, most patients expressed guilt cognitions of “failing maternal responsibilities” due to their inability to fully participate in children’s academic supervision, companionship, and psychological guidance because of treatment. Some patients deliberately concealed their illness and suppressed negative emotions for fear of affecting their children. However, adolescent children are emotionally perceptive and can readily detect maternal abnormalities without being able to communicate effectively, ultimately creating a state of “communication freezing” characterised by mutual avoidance and emotional estrangement [36], which in turn induces persistent maternal guilt and role frustration. This micro-mechanism has been rarely reported in previous studies. The core of intervention for these patients is cognitive restructuring and communication rebuilding: helping patients recognise and adjust excessively high self-expectations, accepting the legitimacy of “fulfilling motherhood differently under treatment constraints”; simultaneously, professional guidance could help patients communicate illness and emotional needs with their children in age-appropriate ways, breaking the “communication freezing” with adolescent children.

Although both role overload and role disablement types presented severe levels of parenting concerns, their core difficulties differed fundamentally. The role overload type originated from “excessive load”—patients had the willingness and ability to fulfill maternal responsibilities, but physical fatigue rendered them unable to do so. The role disablement type, in contrast, originated from “resource exhaustion”—comprehensive deficits in economic, human, and information support meant that even when patients’ role expectations were not high, role adaptation remained unattainable. This indicates that the availability of structural resources is a prerequisite for achieving role adaptation. In the complete absence of resources, psychological intervention or cognitive adjustment alone cannot produce substantial effects [37]. From a clinical intervention perspective, patients with the role disablement type require external systems to inject resources. Specifically, interventions can be approached from three aspects: at the economic level, healthcare providers could conduct financial assessments and guide patients to access institutional grants, charitable assistance, and public welfare benefits to alleviate the strain of treatment costs on parenting resources; at the human resources level, social workers could assist in linking community childcare, volunteer support, and patient advocacy organisations as alternative care resources to address the deficit in family caregiving; at the information level, disease knowledge, treatment options, and parent–child communication guidance could be provided in accessible language, using psychoeducational programmes or parenting support programmes to help patients acquire practical skills for managing role changes.

Beyond the negative role conflict subtypes, the role resilience type identified in this study provides a positive disease adaptation breakthrough for clinical intervention. This group of patients could reconstruct the meaning of maternal roles, shift parenting patterns from task-oriented care to emotional companionship, and proactively engage in cognitive adjustment and behavioural adaptation, effectively alleviating role conflict and parenting stress. This finding resonates with the “post-traumatic growth” theory proposed by Senger et al. [38]. However, our study confirms that patients’ positive adaptation does not merely reflect psychological resilience; its core lies in flexible and adjustable role expectations and proactive role reconstruction behaviours. The role resilience type suggests an intervention pathway: clinicians can facilitate the transformation of other subtypes towards role resilience by helping patients identify and adjust their standards of a “good mother” and guiding them from task-oriented to emotionally oriented parenting models. This provides a clear direction for stratified interventions.

The indigenous Chinese family structure constitutes the core contextual backdrop for patients’ parenting dilemmas. Currently, nuclear families have become the predominant pattern among married households in China [39]. Daily childcare, academic supervision, and emotional nurturing are largely undertaken by mothers, while spouses primarily assume the role of breadwinners, with paternal involvement in childcare being notably absent [40]. Simultaneously, the advantages of traditional extended families featuring multigenerational cohabitation and mutual support are progressively diminishing. Most families face practical constraints such as grandparents residing in different locations, advanced age, or insufficient energy [41], making it difficult to obtain stable and sustainable intergenerational compensatory care, resulting in a severe shortage of effective intergenerational support. Within this familial context, the tolerance for childcare inadequacy is substantially reduced; once maternal illness impairs childcare capacity, the existing family care chain is prone to rupture without buffering or compensatory mechanisms, ultimately exacerbating patients’ parenting stress and psychological distress. Given the current clinical landscape in Chinese oncology settings, parenting stress and family support status should be incorporated into routine screening at initial diagnosis and each treatment cycle to identify high-risk patients vulnerable to non-adherence at the earliest possible stage.

In summary, parenting concerns among breast cancer patients are not merely negative emotions but result from role imbalance driven by the interplay of multiple role expectations, physical burden, family resources, and adaptive behaviours. Therefore, clinical interventions should not be limited to general psychological support but should deliver stratified, targeted interventions according to different subtypes. Based on a four-dimensional analytical framework encompassing role expectation, role load, role resources, and role adaptation, this study moves beyond Western unidimensional models of role conflict and, by incorporating the characteristics of Chinese family structures, reveals the foundational role of structural resources in maternal role adaptation and further identifies role resilience as a positive adaptive pathway. This typology not only enriches the application of social role theory in chronic illness care but also provides actionable, scalable stratified intervention strategies for oncology clinical practice, which may help improve treatment adherence among young breast cancer mothers and optimise their long-term psychological adaptation and overall recovery outcomes.

### 4.1. Strengths and Limitations

Purposive sampling of patients from the Department of Breast Surgery, the Day Ward, and the Breast Surgery Outpatient Clinic was conducted to ensure sample diversity and representativeness. During data collection, a multi-channel approach combining qualitative interviews and electronic medical records was used, enriching the data. The findings are presented in the form of user personas, which intuitively demonstrate the heterogeneity of patients’ parenting concerns.

However, this study has several limitations. First, the sample comprised only married women, and the parenting concerns of divorced or single mothers with breast cancer have not been addressed, limiting the generalisability of the findings. Second, the inherent subjectivity of qualitative research is unavoidable; although we controlled for subjectivity through investigator triangulation, continuous reflexivity, and an audit trail, the identification and labelling of the four personas remain inevitably influenced by the researchers’ interpretive framework.

### 4.2. Implications and Recommendations for Further Research

Based on the findings and limitations of this study, future research may be advanced in two directions. First, subsequent studies should include divorced, widowed, or single-parent households to compare parenting concerns across different family structures, while also adopting longitudinal designs to track persona transitions over time, informing dynamic interventions aligned with the illness trajectory. Second, as this study is a small-sample, single-centre qualitative study, future research should develop assessment scales or classification algorithms to validate the stability and discriminant validity of the four personas in larger samples, and on this basis design tailored interventions targeting each persona’s core difficulties, with their clinical effectiveness evaluated through randomised controlled trials—thus moving the persona model from description to application.

## 5. Conclusions

Grounded in social role theory, this study constructed four heterogeneous parenting concern personas among breast cancer patients, highlighting the important value of stratified precision strategies and individualised support in clinical nursing practice. Future research could conduct large-sample empirical studies to further validate the stability and applicability of the persona model, and continuously optimise persona-based stratified intervention protocols, with the aim of precisely reducing parenting concern levels across different subtypes and improving patients’ maternal adaptation status and parenting-related quality of life.

## Figures and Tables

**Table 1:** Interview Guide for Parenting Concerns in Breast Cancer Patients.

No.	Question
1	Compared with before diagnosis, do you have any new thoughts about your role as a “mother” since your diagnosis?
2	After being diagnosed with breast cancer, how do you feel inside when you continue to care for your minor children?
3	How do your family members’ attitudes toward your dual roles as “patient” and “mother” affect your psychological experience?
4	What difficulties have you encountered in participating in your children’s daily care during treatment?
5	In facing your children’s needs, what situations do you currently find difficult to manage?
6	What challenges have you encountered in the division of parenting responsibilities?
7	When facing difficulties, how do you cope?

**Table 2:** Participant Characteristics.

ID	Age (Years)	Marital Status	Educational Level	Employment Status	Residence	Disease Duration (Months)	Cancer Stage	Treatment Status	No. of Children (No. of Minor Children)	Family Structure	PCQ Score
P1	45	Married	Middle school	Unemployed	Urban	6	I	Radiotherapy	2 (1)	Nuclear	28
P2	36	Married	University	Unemployed	Urban	2	II	Chemotherapy	2 (2)	Nuclear	51
P3	40	Married	High school	Employed	Urban	8	I	Post-surgery	2 (2)	Nuclear	30
P4	39	Married	University	Employed	Urban	1	I	Chemotherapy	2 (2)	Nuclear	50
P5	34	Married	High school	Unemployed	Rural	3	II	Pre-surgery	2 (2)	Nuclear	55
P6	29	Married	Middle school	Unemployed	Rural	3	IV	Pre-surgery	1 (1)	Nuclear	64
P7	32	Married	High school	Unemployed	Rural	5	II	Post-surgery	1 (1)	Nuclear	56
P8	43	Married	University	Unemployed	Urban	10	III	Chemotherapy	1 (1)	Stem	33
P9	33	Married	Middle school	Employed	Urban	13	I	Post-surgery	3 (3)	Stem	61
P10	44	Married	Middle school	Unemployed	Urban	9	II	Chemotherapy	2 (2)	Nuclear	47
P11	35	Married	High school	Unemployed	Rural	1	III	Pre-surgery	2 (2)	Stem	57
P12	37	Married	Middle school	Unemployed	Rural	13	II	Post-surgery	2 (2)	Stem	44
P13	34	Married	University	Employed	Urban	12	I	Chemotherapy	1 (1)	Stem	41
P14	32	Married	University	Unemployed	Urban	9	II	Chemotherapy	1 (1)	Stem	45
P15	28	Married	University	Unemployed	Urban	4	III	Pre-surgery	1 (1)	Stem	53
P16	26	Married	University	Employed	Urban	7	III	Post-surgery	1 (1)	Nuclear	51
P17	29	Married	High school	Unemployed	Rural	2	II	Pre-surgery	1 (1)	Nuclear	52

Note: Nuclear family: couple living with minor children; stem family: two or more generations of direct lineage living together.

**Table 3:** Characteristics of Four Parenting Concern Personas Based on Social Role Theory.

Persona Type	Role Overload	Role Strain	Role Resilience	Role Disablement
Persona	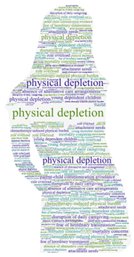	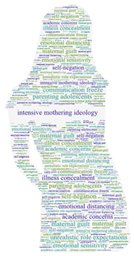	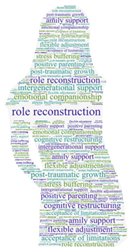	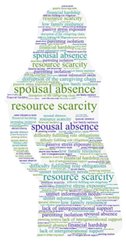
Individuals	P02, P04, P05, P07, P15, P16, P17	P10, P12, P13, P14	P01, P03, P08	P06, P09, P11
PCQ Mean	52.6	44.3	30.3	60.7
Age (years)	26–39 years (mean: 32.0 ± 4.7 years)	32–44 years ( mean: 36.8 ± 5.3 years)	43–45 years (mean: 42.7 ± 2.5 years)	29–35 years (mean: 32.3 ± 3.1 years)
Disease duration (months)	2–7 months	9–13 months	9–13 months	1–3 months
Age of minor children (years)	1–13 years	10–18 years	14–18 years	13 months–8 years
Educational level	High school, University	Middle school, University	Middle school, High school	Middle school, High school
Place of residence	Rural/Urban (predominantly urban)	Rural/Urban (predominantly urban)	Urban	Rural/Urban (predominantly urban)
Family structure	Predominantly nuclear	Predominantly stem	Predominantly nuclear	Predominantly stem
Family support level	Spouse engaged in manual labour with insufficient daytime care; lack of long-term intergenerational support from grandparents; weak family human resources buffer.	Spouse provides basic daily care but insufficient attention to patients’ psychological/emotional needs and adolescent parenting guidance needs.	Spouse and grandparents share parenting and rehabilitation care; fully empathise with patients’ psychological needs; adequate intergenerational support.	Spouse engaged in long-term migrant work or business travel with chronic absence; no grandparental assistance; additionally needs to care for elderly parents; dual deficits in human and emotional support.
Role expectation	Conventional maternal standards, no excessive self-expectations	Internalised perfect mother cognition; extremely high self-expectations regarding children’s academic and psychological guidance	Flexibly adjusts maternal standards; accepts that “limited companionship is still a qualified mother”	Normal maternal expectations, no extreme perfectionism
Role load	Acute-phase chemotherapy exhaustion; high-intensity daily care tasks for young children superimposed; severe deficiency in objective role performance capacity	Moderate physical treatment burden; high emotional and academic parenting needs of adolescent children	Moderate treatment burden; high academic pressure from adolescent children (entrance exams)	Short-term treatment impact; persistent care demands of young children
Role resources	Low income; spouse in manual labour with no time for care; weak intergenerational support; absence of family human resources buffer	Moderate family income; spouse provides basic care but neglects patients’ psychological/emotional needs	Middle to high income; complete family support; spouse and grandparents share parenting tasks	Low income; spouse chronically absent; no intergenerational care support; comprehensive deficits in economic, human, and information resources
Role adaptation	Passive avoidance; unable to engage in high-quality parent–child interaction; prone to parent–child estrangement	Selective self-disclosure; persistent self-negation; prone to communication freezing	Proactive reconstruction of maternal role; shifts from task-oriented care to emotional companionship; actively integrates family resources	Passive coping; no access to parenting or disease-related support information; unable to actively regulate parent–child relationship
Core parenting concerns	1. Fear of disease progression and genetic transmission; unable to accompany children into adulthood 2. Severe energy depletion after chemotherapy; unable to manage daily care of young children 3. Concern that young children will develop self-care and psychological deficits due to mother’s absence 4. Lack of cancer-specific parenting guidance; uncertain how to balance treatment and childcare	1. Own illness creates psychological burden for adolescent children, interfering with academics and social life 2. Unable to participate in adolescent psychological guidance; feels has failed maternal responsibility 3. Long-term treatment prevents providing stable support for children’s growth; uncertain about the future 4. Worries about cancer genetic risk affecting children’s lifelong health	1. Concerns about children’s independent living skills and long-term development 2. Worries about disease inheritance harming children’s physical and mental health 3. Illness interferes with children’s critical academic transition stages (middle/high school entrance exams) 4. Unable to share practical parenting pressures such as exam preparation and academic planning	1. Own illness renders her unable to care for young children, with no family members to substitute 2. Limited family income cannot support both children’s education and own treatment costs 3. Lack of disease and parenting information channels; no external support resources 4. Unable to balance treatment/rehabilitation with parenting responsibilities; no avenues for help
Primary coping strategies	Passive avoidance	Selective self-disclosure	Active coping	Passive coping

Note: Nuclear family: couple living with minor children; stem family: two or more generations of direct lineage living together. The word clouds in the “Persona” row were generated using WordArt (https://wordart.com), with keywords manually extracted from the coding labels under each analytical dimension. Keywords for each persona included: Role Overload—physical exhaustion, chemotherapy burden, young children, caregiving obstruction, attachment needs, death worry, lack of caregivers, parent–child avoidance, genetic fear, role overload; Role Strain—perfect motherhood, adolescent nurturing, psychological guilt, excessive role expectations, communication freezing, emotional estrangement, illness concealment, academic worry, self-negation, emotional sensitivity; Role Resilience—role reconstruction, emotional companionship, flexible adjustment, cognitive restructuring, family support, intergenerational assistance, post-traumatic growth, accepting limitations, positive parenting, stress buffering; Role Disablement—resource depletion, spousal absence, lack of intergenerational support, financial strain, information deficit, parenting isolation, caregiving incapacity, low family tolerance, passive endurance, caregiving chain rupture. The font size of each term reflects its relative frequency in the coded data.

## Data Availability

The raw data supporting the conclusions of this article will be made available by the authors on request.

## Supplementary Materials

The following supporting information can be downloaded at: https://www.mdpi.com/article/10.3390/curroncol33090501/s1, Supplementary Materials S1: Consolidated Criteria for Reporting Qualitative Research (COREQ): 32-item checklist.
