# Supplementary material for: Breast Cancer Patients’ Parenting Concerns User Personas: A Qualitative Analysis
Source: Curr Oncol. 2026 Aug 24;33(9):501. doi: 10.3390/curroncol33090501 (PMC13605563; doi:10.3390/curroncol33090501)
Supplement: Supplementary file 1 [file curroncol-33-00501-s001.zip › curroncol-4476834-supplementary.pdf]

Supplementary Materials S1: Consolidated Criteria for Reporting Qualitative Research (COREQ): 32-item checklist

1.Which author(s) conducted the interview or focus group?	Location in the manuscript: [Page 6, Section 2.4 Data Collection]<br>"All interviews were conducted by a researcher (Ying Zhang) trained in qualitative research methods."
2.What were the researcher's credentials? (e.g. PhD, MD)	Location in the manuscript: [Pages 8-9, Section 2.6 Rigour and Reflexivity]<br>"The research team comprised one expert with over 20 years of experience in psychosocial oncology (Jingfang Hong, PhD), one expert trained in systematic qualitative research methodology (Yin Wang, PhD), and four master's-level nursing students trained in qualitative research methods (Ying Zhang, Ming Liu , Xiaoning Fan, Hailing Tu)."
3.What was their occupation at the time of the study?	Location in the manuscript: [Pages 8-9, Section 2.6 Rigour and Reflexivity]<br>"The research team comprised one expert with over 20 years of experience in psychosocial oncology (Jingfang Hong, PhD), one expert trained in systematic qualitative research methodology (Yin Wang, PhD), and four master's-level nursing students trained in qualitative research methods (Ying Zhang, Ming Liu , Xiaoning Fan, Hailing Tu)."
4.Was the researcher male or female?	Location in the manuscript: [Page 9, 2.6 Rigour and Reflexivity]<br>"All team members were female"
5.What experience or training did the researcher have?	Location in the manuscript: [Pages 8-9, Section 2.6 Rigour and Reflexivity]<br>"The research team comprised one expert with over 20 years of experience in psychosocial oncology (Jingfang Hong, PhD), one expert trained in systematic qualitative research methodology (Yin Wang, PhD), and four master's-level nursing students trained in qualitative research methods (Ying Zhang, Ming Liu , Xiaoning Fan, Hailing Tu)."
6.Was a relationship established prior to study commencement?	Location in the manuscript: [Page 9, Section 2.6 Rigour and Reflexivity]<br>"interviewers had no prior caregiving relationships with participants."
7.What did the participants know about the researcher?	1.Location in the manuscript: [Page 5, Section 2.1 Ethical Considerations]<br>"All participants received written and verbal information regarding the study purpose, procedures, the voluntary nature of participation, and their right to withdraw at any time prior to enrollment, and all provided written informed consent."<br><br>2.Location in the manuscript: [Page 5, Section 2.6 Rigour and Reflexivity]<br>"and interviewers had no prior caregiving relationships with participants"

8.What characteristics were reported about the interviewer/facilitator?	Location in the manuscript: [Pages 8-9, Section 2.6 Rigour and Reflexivity]<br>The gender, professional backgrounds, and relationship with participants of the team members have all been reported.
9.What methodological orientation underpinned the study?	1.Location in the manuscript: [Page 4,Section 1.Introduction]："Social role theory"<br><br>2.Location in the manuscript: [Page 5,Section 2.2 Study Design]:"A descriptive qualitative study design was adopted to achieve the research objectives."
10.How were participants selected?	Location in the manuscript: [Page 5, Section 2.3 Participant Recruitment]<br>"purposive sampling following the principle of maximum variation was employed to recruit participants"
11.How were participants approached?	Location in the manuscript: [Page 5, Section 2.3 Participant Recruitment]<br>"Potential participants meeting the inclusion criteria were identified through electronic medical record review, and eligible patients were subsequently provided with study information and invitations to enroll."
12.How many participants were in the study?	Location in the manuscript: [Page 9, Section 3.1 Characteristics of Participants]<br>"A total of 17 participants completed the interviews."
13.How many people refused or dropped out? Reasons?	Location in the manuscript: [Page 6, Section 2.3 Participant Recruitment]<br>"A total of 20 eligible participants were recruited, of whom 3 declined to participate (1 due to low mood, 2 due to physical discomfort)."
14.Where was the data collected?	Location in the manuscript: [Page 6, Section 2.4 Data Collection]<br>"was conducted in a quiet, uninterrupted conference room."
15.Was anyone else present besides participants and researchers?	Location in the manuscript: [Page 6, Section 2.4 Data Collection]<br>"was conducted in a quiet, uninterrupted conference room." No other persons were present.
16.What are the important characteristics of the sample?	Location in the manuscript:<br>Section 3.1 Characteristics of Participants; Table 2
17.Were questions or guides provided? Was it pilot tested?	Location in the manuscript: [Page 6 , Section 2.4 Data Collection]<br>"An initial interview guide was developed through literature review and consultation with oncology experts, and refined through pilot interviews with three participants, resulting in the final interview guide (Table 1)."
18.Were repeat interviews carried out?	Location in the manuscript: [Page 9 , Section 3.1 Characteristics of Participants]<br>"A total of 17 participants completed the interview, with each participant being interviewed once."

19.Did the research use audio or visual recording?	Location in the manuscript: [Page 6 , Section 2.4 Data Collection] “Interviews were audio-recorded throughout”
20.Were field notes made during or after interviews?	Location in the manuscript: [Page 8 , Section 2.6 Rigour and Reflexivity] “Interview data were cross-validated with field notes taken during the analysis process to contextualize the findings.”
21.What was the duration of the interviews?	Location in the manuscript: [Page 6 , Section 2.4 Data Collection] “Each interview lasted 30–60 minutes and was conducted in a quiet, uninterrupted conference room.”
22.Was data saturation discussed?	Location in the manuscript: [Page 5, Section 2.3 Participant Recruitment] “Sample size was determined by data saturation; recruitment ceased when interview data began to repeat and no new themes emerged[27].”
23.Were transcripts returned to participants for comment?	Location in the manuscript: [Page 8 , Section 2.6 Rigour and Reflexivity] “Additionally, transcripts were returned to participants for confirmation after interviews (member checking) to ensure that the analytical results were consistent with participants‘ authentic experiences.”
24.How many data coders coded the data?	Location in the manuscript: [Page 6 , Section 2.5 Data Analysis] “Two researchers (Ying Zhang and Ming Liu) used NVivo 12 software to extract meaning units related to parenting concerns and assigned preliminary codes.”
25.Was a description of the coding tree provided?	Location in the manuscript: [Pages 6-7 , Section 2.5 Data Analysis] A description of the coding structure, from sub-themes to the four core analytical dimensions, is provided in the Methods section.
26.Were themes identified in advance or derived from the data?	Location in the manuscript: [Pages 6-7 , Section 2.5 Data Analysis] Theory-guided dimensions (social role theory) were combined with inductive coding from the data, using both deductive and inductive strategies.
27.What software was used to manage the data?	Location in the manuscript: [Page 6 , Section 2.5 Data Analysis] “...... used NVivo 12 software to extract meaning units related to parenting concerns and assigned preliminary codes.”
28.Did participants provide feedback on the findings?	Location in the manuscript: [Page 8, Section 2.6 Rigour and Reflexivity] “Additionally, transcripts were returned to participants for confirmation after interviews (member checking) to ensure that the analytical results were consistent with participants‘ authentic experiences.”

29.Were participant quotations presented and identified?	Location in the manuscript: [Pages 9-13 , Section 3.2 User Persona Labelling System for Parenting Concerns in Breast Cancer Patients]<br>Each persona was illustrated with representative quotations, with participants identified by their IDs (e.g., P02, P05, etc.).
30.Was there consistency between data and findings?	Location in the manuscript: [Pages 9-13, Section 3 Results]<br>The findings are consistent with the interview data, and the quotations adequately support the characteristics of each persona.
31.Were major themes clearly presented?	Location in the manuscript: Table 3<br>The key characteristics of the four personas are clearly presented.
32.Was there a description of minor themes or diverse cases?	1.Location in the manuscript: Table 3<br>Each persona is presented with key characteristics, a narrative summary, and representative quotations.<br><br>2.Location in the manuscript: [Page 17 ,Section 4.1 Strengths and Limitations]<br>It is noted that divorced or single mothers were not included in the sample.<br>"First, the sample comprised only married women, and the parenting concerns of divorced or single mothers with breast cancer have not been addressed, limiting the generalisability of the findings."
